# Identification of differentially expressed miRNAs and their target genes in response to brassinolide treatment on flowering of tree peony (*Paeonia ostii*)

**DOI:** 10.1080/15592324.2022.2056364

**Published:** 2022-03-27

**Authors:** Lin Zhang, Chengwei Song, Dalong Guo, Lili Guo, Xiaogai Hou, Huafang Wang

**Affiliations:** a College of Biological Sciences and Technology, Beijing Forestry University, Beijing, China; b College of Agriculture/Tree Peony, Henan University of Science and Technology, Luoyang, Henan sheng, China; c College of Horticulture and Plant Protection, Henan University of Science and Technology, Luoyang, Henan sheng, China

**Keywords:** Brassinolide, flower development, microRNA, *Paeonia ostii*, transcriptome

## Abstract

Tree peony is a famous flower plant in China, but the short and concentrated flowering period limits its ornamental value and economic value. Brassinolide (BR) plays an important role in plant growth and development including flowering. There have been a large number of reports on the molecular aspects of the flowering process, but the genetic mechanism that was responsible for miRNA-guided regulation of tree peony is almost unclear. In this study, the leaves of tree peony cultivar, ‘Feng Dan’, were sprayed with different concentrations of BR, and the obvious bloom delay was found at the treatment with BR 50 μg/L. The small RNA sequencing and transcriptome sequencing were performed on the petals of tree peony under an untreated control (CK) and the treatment with BR 50 μg/L during four consecutive flowering development stages. A total of 22 known miRNAs belonging to 12 families were identified and 84 novel miRNAs were predicted. Combined with transcriptome data, a total of 376 target genes were predicted for the 18 differentially expressed known miRNAs and 177 target genes were predicted for the 23 differentially expressed novel miRNAs. Additionally, the potential miRNAs and their target genes were identified, including miR156b targeting *SPL*, miR172a_4 targeting *AP2* and four novel miRNAs targeting *SPA1*, and revealed that they might affect the flowering time in tree peony. Collectively, these results would provide a theoretical basis for further analysis of miRNA-guided regulation on flowering period in tree peony.

## Introduction

Tree peony is a perennial woody deciduous shrub, belonging to the family *Paeoniaceae*, section Moutan, and genus *Paeonia*,^1^ and it has been renowned for its large flowers, diverse flower color, graceful appearance, and strong fragrance.^2,3^ Tree peony has a long history of ornamental cultivation, and with long-term natural and artificial selection, there are more than 2000 varieties that vary in flowering traits of tree peony worldwide.^4,5^ The natural flowering period of tree peony is concentrated and short. Single flowers last for 3–5 days in some genotypes and for 7–10 days in other genotypes, while the overall duration of flowering in the whole plant is 20–30 days.^2^ Although there are many varieties of tree peony, most varieties exhibit medium flowering period traits and the number of early and late flowering varieties is small. These parameters directly affect the ornamental and economic values of tree peony.

Plant hormones play an important role in regulating flowering and have been extensively studied in the model plant Arabidopsis.^6^ In the past decade, some studies have been carried out on the regulation of tree peony flowering by spraying hormones, such as GA_3_ and so on.^7^ However, the study of BR regulation of the flowering period of tree peony has not been reported yet. BR, a steroidal plant hormone originally isolated from the pollen of rape (*Brassica napus* L.), plays an important role in the growth and development of plant organs.^8^ Plants treated with exogenous BR could not only improve their resistance to temperature, drought, water tolerance, salt, heavy metals, and other abiotic stresses but also regulate the timing of plant flowering.^9,10^ BR affects the flowering period and ornamental quality in *chrysanthemum* at different growth and development stages, postponing its flowering time by 3–4 days.^11^ Spraying different concentrations of BR solution at the initial bud stage in honeysuckle can postpone flowering time by 2–6 days.^12^ Recently, it was found that BR inhibits flowering by promoting the expression of *FLC* and its homologous genes during *Arabidopsis thaliana* growth and development.^10^ Many studies have been conducted on the effect of BR on growth and development in tree peony, but no study has been conducted on the effect of BR on the regulation of flowering time.^13,14^

To analyze the molecular mechanism of tree peony flowering regulation, many research projects have been carried out, especially after the publication of the draft genome of *Paeonia suffruticosa*.^15^ A normalized cDNA library from flower buds of tree peony during chilling fulfillment was sequenced on a Roche 454 GS FLX platform. *De novo* assembly yielded 23,652 contigs and singletons, providing a significant resource for gene discovery related to endodormancy.^16^ Based on Illumina-based *de novo* transcriptome sequencing, 291 unigene sequences associated with early and late flowering were identified in tree peony by bulked segregant RNA-seq (BSR-seq).^17^ Subsequently, a high-density genetic map was constructed from interspecific F1 population of *P. ostii* ‘Fengdan Bai’ and *P. suffruticosa* ‘Xin Riyuejin’ using a genotyping-by-sequencing (GBS) approach.^18^ Based on this map, one QTL associated with flowering period was detected that explained 20.4% of the phenotypic variance in flowering period. A total of 64 flowering-related genes were identified in tree peony flower buds using 454-based transcriptome sequencing technology, and a model of the genetic regulation network for flowering induction pathways and floral organ development was constructed.^5^ Several genes associated with flowering have also been cloned in tree peony, such as GA biosynthesis and signaling genes, SQUAMOSA PROMOTER BINDING PROTEIN-LIKE (SPL) transcription factors.^19,20^ The mechanism regulating flowering period in tree peony has not been fully elucidated.^17^ A better understanding of the molecular mechanism of flowering time in tree peony could provide a theoretical basis for manipulating flowering time and duration.

Small RNAs, mainly including microRNAs (miRNAs), Piwi-interacting RNAs (piRNAs), and short-interfering RNAs (siRNAs), are an important class of regulatory molecules. They mainly function in inducing gene silencing, post-transcriptional regulation of genes, and in general play a regulatory role in cell growth, differentiation, and a variety of biological processes.^21–23^ A growing body of evidence indicates that miRNAs function as key regulators of flowering time in plants.^24^ Collective studies have demonstrated that miRNAs regulate flowering time, floral morphology, petal number, inflorescence development, male and female fertility, the differentiation of apical meristematic cells, and other processes.^25^ miR172 affected the flowering time,^26^ floral organ properties,^27^ and flower certainty^28^ by regulating the expression of an *AP2*-like gene. Using an activation-tagging approach, miR172 was shown to induce early flowering and disrupt the specification of floral organ identity when overexpressed in *A. thaliana*.^26^ miR156 and miR157 were found to prevent early flowering mainly by inhibiting the translation of *SPL3* mRNA and that the levels of miR156 and miR157 were lowest during adult leaf and inflorescence growth, which enabled SPL3 and other genes to accumulate and regulate flower formation.^29^ Twelve conserved miRNAs and 18 novel miRNAs were reported to exhibit significant changes in response to copper stress in *Paeonia ostia*.^30^ A total of 32 conserved miRNAs and 17 putative novel miRNAs were identified in tree peony buds during chilling-induced dormancy release.^31^ High-throughput sequencing technology was used to identify miRNAs and lncRNAs in the seeds of two tree peony cultivars that significantly differ in their level of α-linolenic acid.^32^ As a result, 9 miRNAs and 39 lncRNAs were predicted to target lipid-related genes in developing seeds. In another study, transcriptome, small RNA, and degradome sequencing were employed to systematically investigate miRNAs in *Paeonia ostii*, resulting in the identification of 113 predicted novel miRNA hairpin precursors.^33^ Although small RNAs have been extensively studied in model plant systems, little is known about their identity and function in tree peony, especially the potential role of small RNAs in the regulation of flowering time mechanisms in tree peony remain unclear.

In the present study, we used *Paeonia ostii* T. Hong et J. X. Zhang var. *lishizhenenii* B. A. Shen as material, which has been widely cultivated and used in China and named ‘Feng Dan’. By spraying different concentrations of BR to explore the effect of BR on flowering period of peony. Small RNA sequencing and transcriptome sequencing were used to identify miRNAs and predict target genes of flowering period in ‘Feng Dan’ at control group and BR 50 μg/L treatment at four consecutive flowering development stages and 22 known miRNAs and 84 novel miRNAs were identified and predicted. The potential interaction between miRNAs and target genes regulating flowering time was also suggested, including four novel miRNAs targeting *SPA1*, miR156b targeting *SPL*, and miR172 targeting *AP2*. This study would provide a theoretical basis for understanding the role of small RNA in regulating flowering time in tree peony.

## Materials and methods

### Plant materials

Seven-year-old tree peony plants (*Paeonia ostii* T. Hong et J. X. Zhang var. *lishizhenenii* B. A. Shen) were used as material, which were planted and grown in an experimental field at Henan University of Science and Technology (Luoyang, China). The leaves of the tree peony plants were sprayed with BR 25 μg/L, 50 μg/L, 100 μg/L, and 200 μg/L at April 3, when the tree peony was at the flowering bud stage (Treatment). Control plants (CK) were sprayed with double distilled water (DD water). The date of bud burst, flower opening, full bloom, and petal decay were recorded for both the BR treatment and CK groups.

### Total RNA extraction

**Figure 1.**
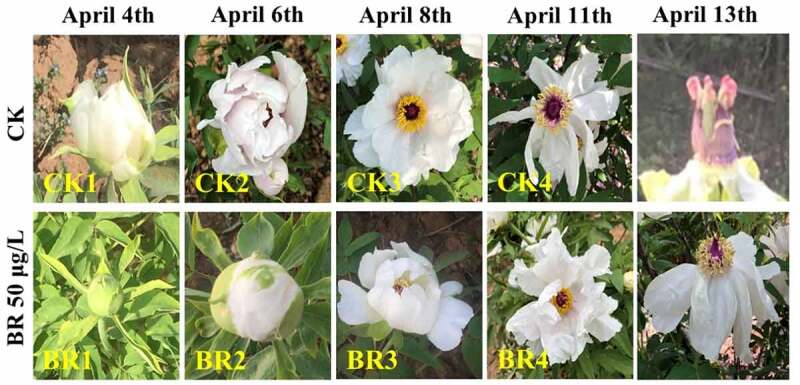
The flowering developmental stages in CK and BR 50 μg/L treatment.

Petals in both treatment with BR 50 μg/L and CK plant groups were collected at bud burst (CK1 and BR1), flower opening (CK2 and BR2), full bloom (CK3 and BR3), and post-bloom when petals were wilting and breaking down (CK4 and BR4) (Figure 1). Each petal samples were collected and pooled from at least five plants with same genetic background and similar growth rates. Three biological replicates were taken at each developmental stage for both BR treatment group and CK group. All of the samples were immediately frozen in liquid nitrogen as collected and then shifted to an ultra-low temperature refrigerator at −80°C for storage. The total RNA was isolated using a RNAprep Pure Plant Kit (Polysaccharides & Polyphenolics-rich) (Tiangen, Beijing, China) following the manufacturer’s protocol. The purity and quantity of the extracted RNA were determined using a NanoDrop 2000 UV-Vis Spectrophotometer at OD_260/280_ and OD_260/230_, respectively. An Agilent 2100 Bioanalyzer was used to assess the integrity of RNA with quality indicators that included RIN values and 28S/18S ratios.

### Small RNA library construction and sequencing

The total RNA of all samples was subjected to 15% denaturing polyacrylamide gel electrophoresis to separate and purify 18–30 nt RNA fragments. A 5-adenylated and 3-blocked adaptor was then PCR-ligated to the 3’ end of the small RNA fragments. RT primers with unique molecular identifiers (UMI) were added and hybridized with the 3’ linker attached to the RNA and excess free 3’ linker. Then digestive enzyme was used to remove the 3’ linker, and a 5’ linker was connected to the 5’ end. Since the linker is preferentially connected to single-stranded molecules, it did not connect to the hybrid chain of the 3’ linker and RT primer, which greatly reduced self-connection of the linker. RT primers with UMI were used for reverse transcription and extension to synthesize first-strand cDNA, PCR amplification was performed to generate double-strand cDNA. PCR products in the range of 100–120 bp were separated by 15% polyacrylamide gel electrophoresis. After library quantification and pooling cyclization, the resulting libraries were sequenced on a BGI-seq platform (BGI, Shenzhen, China). The number of clean reads in every sample was greater than 20 M.

### Small RNA analysis

Clean reads were selected from the raw sequencing reads by removing low-quality reads, reads with 5’ primer contaminants, reads without 3’ primer, reads without insertions, reads with poly A, and reads shorter than 18 nt. Low quality was defined as sequences with more than four bases whose quality was less than 10 or those that had more than six bases with a quality less than 13. The clean tags were mapped to the *Paeonia suffruticosa* Andr. reference genome using AASRA software except that was used instead of Rfam. The length distribution of clean tags was then summarized. The length distribution analysis enabled an assessment of the compositions of the sample. All small RNAs were mapped to various RNAs. In the annotation information of the different RNAs, some small RNA reads may be mapped to more than one category. To make sure every unique small RNA was mapped to only one category, the following priority rule was used: MiRbase > pirnabank > snoRNA (plant) > Rfam > other sRNA.

### BGISEQ-500 RNA-Seq

The total RNA from all of 24 samples was extracted separately. The total RNA of each sample was first separated with magnetic oligo (dT) beads to enrich for poly A mRNA, and then fragmented using an interrupting buffer. The random hexamer (N6) primer and M-MuLV Reverse Transcriptase (RNase H) were used for reverse transcription to achieve the first-strand cDNA, and the second strand was synthesized using DNA polymerase I and RNaseH. The ends of dscDNA were repaired and phosphorylated at the 5’ end, and the 3’ end formed a sticky end that protruded ‘A’. Subsequently, the dscDNA strands were ligated with adapters that had a sticky ‘T’ at the 3′ end. The ligation product was amplified using two specific primers. Finally, the PCR product was heat-denatured into a single strand, and the single-stranded DNA was cyclized with splint oligo and DNA ligase to obtain a single-stranded circular DNA library. More than 10 Gb of high-quality bases were generated for each library. All libraries were constructed and sequenced on a BGISEQ-500 RNA Seq platform (BGI Shenzhen, China).

### BGISEQ-500 data analysis

After quality control checks, clean data was separated from the raw data by removing the reads containing adaptor sequences, the reads with a content of unknown bases greater than 5%, low quality reads (when the ratio of bases with a quality value below 10 to the total number of bases in the read was greater than 20%). The resulting, high-quality clean reads were then mapped to the *Paeonia suffruticosa* Andr. reference genome using Bowtie2 (v2.2.5) software. The level of gene expression was quantified using Expectation-Maximization (RSEM) (v1.2.8) software and normalized using the Fragments Per Kilobase Million (FPKM) method.

### Small RNAs and target gene prediction

miRA was used to predict novel miRNA by analyzing the characteristic hairpin structure of the miRNA precursor. Piano, which is based on the Support Vector Machine (SVM) algorithm and transposon interaction information, was used to predict piRNAs. siRNA candidates were predicted based on the characteristic structure that the siRNA was a 22–24 nt double-strand RNA, each strand of which was 2 nt longer than the other stand. Multiple types of software, including psRobot, TAPIR, and TargetFinder, were used to identify potential targets.

### Differential expression analyses of miRNAs and functional enrichment analyses of their target genes

The different expression miRNAs was defined when the read number fold change ≥2 and Q-value ≤0.001. To annotate gene functions, all target genes were aligned against the Kyoto Encyclopedia of Genes (KEGG) and Gene Ontology (GO) database. GO enrichment analysis and KEGG enrichment analysis of target genes were performed using phyper, a function within R software. For the gene matched to multiple protein sequences, the protein with the highest similarity score was considered as the optimal annotation. The P-value was corrected using the Bonferroni method, and a corrected P-value ≤0.05 was used as a threshold. Over-presented GO terms were identified using a hypergeometric test with a significance threshold of 0.05 after the Benjamini and Hochberg FDR correction. KEGG enrichment analysis of the DEGs was performed using the KOBAS 2.0 software.

### Confirmation of mature miRNAs expression

Total RNAs were reverse-transcribed to cDNA using a One Step PrimeScript miRNA cDNA Synthesis Kit (TaKaRa) following the manufacturer’s protocol. SYBR Premix Ex Tag II (TaKaRa) was used for qRT-PCR. Small nuclear RNA U6 was used as an internal reference. *Actin* was used as an internal reference for expression of target genes. qRT-PCR analyses were conducted using a real-time PCR system (Eppendorf, Hamburg, Germany). The primers of qRT-PCR for miRNAs and target genes are shown in Table S1. 2^−ΔΔCt^ method was used to calculate the relative expression of miRNAs and target genes.

## Results

### Investigation and analysis of flowering period

To determine whether BR has an effect on the flowering period of tree peony, the different stages of flowering were observed and recorded. Under the natural growth conditions (CK), the flowering period of ‘Feng Dan’ lasted 7 days with the flower opening stage occurred on April 6 and the withering stage occurred on April 13. In the treatment groups of BR 100 μg/L and 200 μg/L, tree peony showed faintly shorter flowering period and delayed flowering, with the flower opening stage occurring on April 8 and withering stage occurring on April 14 compared to CK. There was no change in the flowering period of tree peony treated with BR 25 μg/L compared to CK. Notably, at BR 50 μg/L treatment, the flowering period also lasted for 7 days, but the flower opening stage was delayed up to 3 days with the flower opening stage occurring on April 9 and withering stage occurring on April 16 compared to CK (Figure 1). These suggest that the BR had a direct or indirect regulatory impact on the flowering period in tree peony, especially at a concentration of BR 50 μg/L. Thus, the subsequent researches were carried out with the BR 50 μg/L treatment, and the sampling period is shown in Figure 1.

### High-throughput sequencing of small RNAs

**Figure 2.**
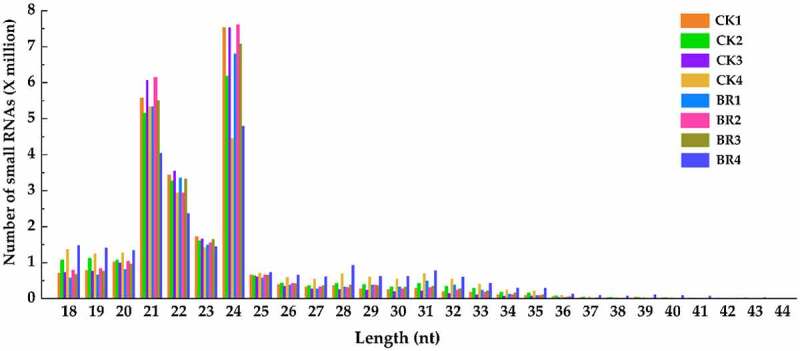
Length distribution of small RNA in CK and BR treatment at four developmental stages. The number after CK and BR indicates the sampling stage of petals collection. CK1 and BR1 indicates bud burst stage. CK2 and BR2 indicates flower opening stage. CK3 and BR3 indicates full bloom stage. CK4 and BR4 indicates post-bloom when petals were wilting and breaking down. The same as below.

A total of 24 samples from the two treatment groups (CK and BR) were sequenced using BGISEQ-500 technology to explore the role of small RNAs in the flowering process of tree peony. The raw read count in independent samples was between 25.0 and 61.5 million reads (Table S2). After removing the low-quality sequences, 3’ or 5’ adaptor sequences, sequences less than 18 nt in length, and sequences containing poly A, the clean read count of each sample was greater than 20 M. The Q20 value in all samples was above 99%, indicating that the quality of the sequencing data was relatively high. The length distribution of small RNAs ranged from 18 to 30 nt, and the type of small RNAs could be roughly determined by their length distribution. A statistical analysis of the length of small RNA sequences indicated that most of the small RNA sequences in each sample were distributed in the 21–24 nt category, in which the largest number of small RNAs were in the 24 nt class, followed by the 21 nt class, which is consistent with the length distribution of small RNAs in plants (Figure 2). After data filtering, clean reads of all the samples were mapped using known, small RNA databases. The mapping percentage ranged from 33.17% to 55.91% (Table S2). The proportion of different types of small RNAs in each sample is presented in Table 1. Many different types of small RNAs were identified, including miRNA (mature), sncRNA, rRNA, snoRNA, tRNA, snRNA, etc. Among them, the number of known miRNAs in samples ranged from 198467 (0.96%) to 636259 (2.61%). The rate of annotation ranged from 39.71% to 66.60%.

**Table 1.** The proportion of different types of small RNAs in each library

Sample	Known miRNA	precursor	rRNA	tRNA	snoRNA	snRNA	Rfam other sncRNA	intergenic	unannotation	Total
CK1_r1	267294 (1.10%)	65362 (0.27%)	670063 (2.76%)	40474 (0.17%)	14689 (0.06%)	5198 (0.02%)	12869 (0.05%)	10041158 (41.39%)	13142037 (54.17%)	24259144 (100%)
CK1_r2	250032 (1.04%)	71740 (0.30%)	450486 (1.87%)	4596 (0.02%)	17303 (0.07%)	4373 (0.02%)	15814 (0.07%)	9728301 (40.34%)	13575221 (56.29%)	24117866 (100%)
CK1_r3	354459 (1.48%)	71153 (0.30%)	268414 (1.12%)	4595 (0.02%)	5644 (0.02%)	2583 (0.01%)	4862 (0.02%)	8337885 (34.86%)	14868562 (62.16%)	23918157 (100%)
CK2_r1	305656 (1.27%)	72880 (0.30%)	224960 (0.93%)	4089 (0.02%)	5314 (0.02%)	4447 (0.02%)	11196 (0.05%)	8762865 (36.28%)	14761155 (61.12%)	24152562 (100%)
CK2_r2	250377 (1.06%)	48252 (0.20%)	360826 (1.52%)	13671 (0.06%)	8597 (0.04%)	5528 (0.02%)	20741 (0.09%)	10214548 (43.14%)	12752871 (53.87%)	23675411 (100%)
CK2_r3	468264 (2.03%)	54305 (0.24%)	613095 (2.66%)	44597 (0.19%)	11375 (0.05%)	5645 (0.02%)	38991 (0.17%)	10011399 (43.47%)	11782710 (61.16%)	23030381 (100%)
CK3_r1	372043 (1.54%)	74063 (0.31%)	454422 (1.89%)	7077 (0.03%)	7499 (0.03%)	3822 (0.02%)	11633 (0.05%)	8611254 (35.75%)	14547609 (60.39%)	24089422 (100%)
CK3_r2	321809 (1.33%)	83000 (0.34%)	366350 (1.52%)	11410 (0.05%)	8276 (0.03%)	4886 (0.02%)	8735 (0.04%)	9326989 (38.66%)	13995904 (58.01%)	24127359 (100%)
CK3_r3	333274 (1.39%)	62927 (0.26%)	210424 (0.88%)	7464 (0.03%)	5124 (0.02%)	2932 (0.01%)	8014 (0.03%)	8047751 (33.56%)	15304533 (63.82%)	23982443 (100%)
CK4_r1	636259 (2.61%)	48298 (0.20%)	425228 (1.75%)	25209 (0.10%)	7141 (0.03%)	5566 (0.02%)	19027 (0.08%)	10460583 (42.96%)	12723968 (52.25%)	24351279 (100%)
CK4_r2	449770 (1.85%)	45321 (0.19%)	664390 (2.74%)	40106 (0.17%)	10070 (0.04%)	8257 (0.03%)	25522 (0.11%)	11334583 (46.70%)	11693006 (48.18%)	24271025 (100%)
CK4_r3	585826 (2.42%)	49521 (0.20%)	716298 (2.96%)	146630 (0.60%)	6819 (0.03%)	6483 (0.03%)	62513 (0.26%)	11141247 (45.96%)	11523301 (47.54%)	24238638 (100%)
BR1_r1	198467 (0.96%)	60571 (0.29%)	239426 (1.16%)	5378 (0.03%)	7328 (0.04%)	7504 (0.04%)	5340 (0.03%)	7482168 (36.12%)	12710239 (61.35%)	20716421 (100%)
BR1_r2	215520 (0.91%)	48505 (0.20%)	194564 (0.82%)	12980 (0.05%)	7461 (0.03%)	6537 (0.03%)	10612 (0.04%)	7450253 (31.32%)	15842426 (66.60%)	23788858 (100%)
BR1_r3	281314 (1.17%)	64405 (0.27%)	186261 (0.78%)	24163 (0.10%)	5591 (0.02%)	8597 (0.04%)	16224 (0.07%)	7473133 (31.13%)	15947043 (66.43%)	24006731 (100%)
BR2_r1	349891 (1.47%)	71914 (0.30%)	493025 (2.07%)	13872 (0.06%)	8211 (0.03%)	5324 (0.02%)	20542 (0.09%)	8954173 (37.54%)	13934034 (58.42%)	23850986 (100%)
BR2_r2	433177 (1.79%)	63283 (0.26%)	337017 (1.40%)	15192 (0.06%)	5101 (0.02%)	4544 (0.02%)	19035 (0.08%)	8605489 (35.64%)	14665310 (60.73%)	24148148 (100%)
BR2_r3	328477 (1.33%)	87496 (0.35%)	761117 (3.08%)	8352 (0.03%)	5755 (0.02%)	5420 (0.02%)	16927 (0.07%)	9294948 (37.65%)	14176331 (57.43%)	24684823 (100%)
BR3_r1	488745 (2.04%)	173596 (0.72%)	801169 (3.34%)	11388 (0.05%)	8139 (0.03%)	4134 (0.02%)	8868 (0.04%)	9202038 (38.35%)	13295913 (55.41%)	23993990 (100%)
BR3_r2	304547 (1.30%)	48126 (0.21%)	433168 (1.85%)	12885 (0.05%)	9010 (0.04%)	6754 (0.03%)	14546 (0.06%)	9411391 (40.13%)	13211624 (56.33%)	23452051 (100%)
BR3_r3	345437 (1.42%)	62604 (0.26%)	584726 (2.40%)	17421 (0.07%)	10454 (0.04%)	4515 (0.02%)	21794 (0.09%)	8952227 (36.81%)	14323502 (58.89%)	24322680 (100%)
BR4_r1	237897 (0.99%)	31908 (0.13%)	629032 (2.62%)	29548 (0.12%)	7487 (0.03%)	5916 (0.02%)	39524 (0.16%)	11258435 (46.95%)	11740919 (48.96%)	23980666 (100%)
BR4_r2	224688 (0.92%)	34058 (0.14%)	664802 (2.71%)	36650 (0.15%)	8260 (0.03%)	6341 (0.03%)	27117 (0.11%)	11840460 (48.28%)	11683555 (47.64%)	24525931 (100%)
BR4_r3	231202 (0.95%)	22555 (0.09%)	716676 (2.93%)	51137 (0.21%)	6067 (0.02%)	8203 (0.03%)	26220 (0.11%)	13659900 (55.94%)	9697808 (39.71%)	24419768 (100%)

### Identification of known and novel miRNAs

**Figure 3.**
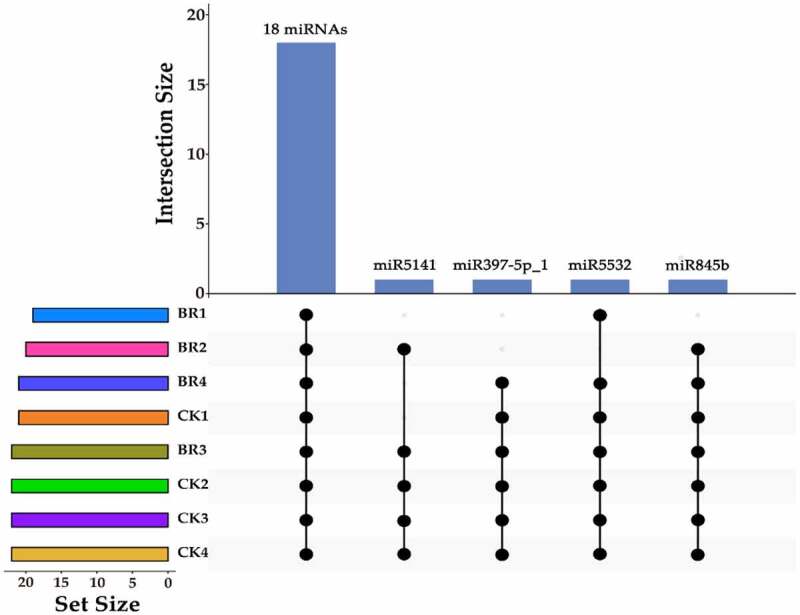
The upset map of known miRNAs in CK and BR treatment at four developmental stages. The left bar chart indicates the total number of miRNAs in each sample. The upper bar chart indicates the number of miRNAs intersections in each sample. The dot at bottom right indicates the specific situation of miRNAs intersection. All of the CK and BR treatment at four developmental stages in tree peony had the same 18 miRNAs.

**Figure 4.**
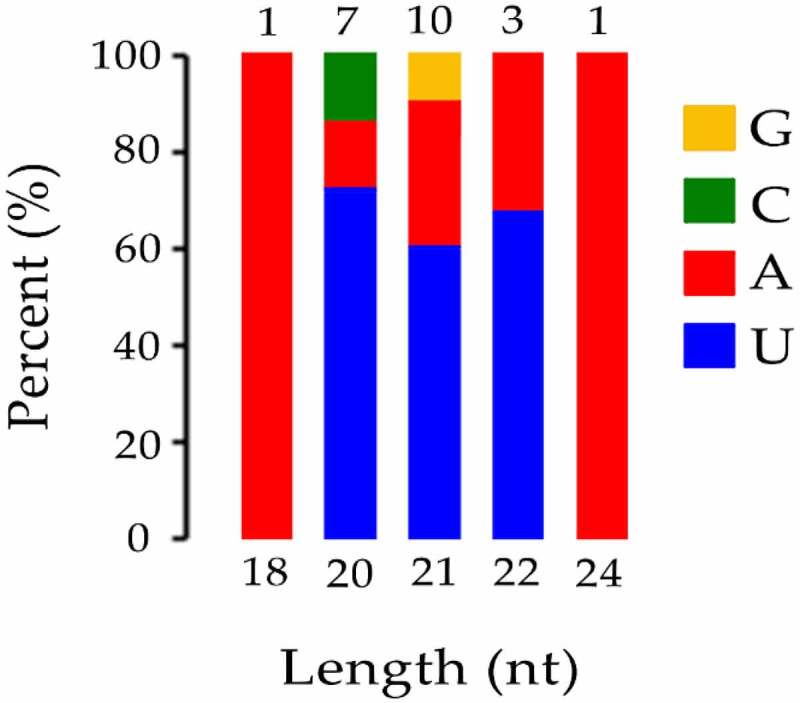
The first base distribution of known miRNA. The X-axis represents the length of miRNAs. The Y-axis represents the proportion of mature miRNAs with a certain base type as the first base. The number on the column chart represents the number of miRNA types of a certain length.

To identify conserved miRNAs of petals in tree peony, clean reads were mapped to reference genomes and other small RNA databases. Two mismatches were allowed when clean data were aligned to with known miRNA. A total of 22 known miRNAs belonging to 12 families were identified in all of the samples collectively (Table S3). Among the 22 known miRNAs, 6, 5, and 2 members were identified as belonging to the miR156, miR396, and miR171 families, respectively. Most miRNA families were represented by a single member, including the miR159, miR172, miR397, miR399, miR4995, miR5141, miR530, miR5332, and miR845 families. In particular, although only one member (miR159a_1) of miR159 family was found, it was the most abundant sequences in all libraries. Results indicated that different members in different miRNA families exhibited highly different expression levels. Among 22 known miRNAs, 18 known miRNAs, miR156a_2, miR156b, miR156c, miR156f, miR156k, miR156z, miR159a_1, miR171a-3p, miR171a-3p_1, miR172a_4, miR396-3p_1, miR396a-3p_2, miR396b, miR396b-3p, miR396b-5p, miR399e, miR4995, and miR530a_2 were identified in all samples (Figure 3). miR5141 was not identified in the bud burst and post-bloom stage of BR treatment and bud burst stage of CK. miR397-5p_1 was not identified in the bud burst and flower opening stage of BR treatment. miR5532 was not identified in the flower opening stage of BR treatment. miR845b was not identified in the bud burst stage of BR treatment. Analysis of the first nucleotide of all known miRNAs that identified from the CK and BR treatment at four developmental stages in tree peony revealed that most of them possessed a uridine (U) at the start of their 5’-ends (Figure 4).

Novel miRNAs were predicted using miRA with strict standards. In total, 84 novel miRNAs were predicted in the tree peony petal libraries collectively (Table S4). The length of the novel miRNAs ranged from 19 to 30 nt. Among the novel miRNAs, those with a length of 24 nt were the most abundant, followed by those that were 30 nt. Notably, the abundance of novel miRNAs was lower than the abundance of most known miRNA families (Table S5). Among all novel miRNAs, the count of novel_mir55 sequence was the most abundant, followed by novel_mir10, novel_mir40, and novel_mir23. In all samples, the number of novel miRNAs expressed ranged from 22 (BR4) to 29 (BR2). A total of 14 novel miRNAs were expressed in all samples.

### Statistical analysis of BGISEQ −500 RNA seq data

Twenty-four cDNA libraries (12 from the CK group and 12 from the BR group) were subjected to high-throughput RNA-Seq. The raw sequences were filtered to remove low-quality reads, linker contamination, and reads with unknown base N content. In total, 1.82 Gb of raw reads were generated by the BGISEQ-500 platform with an average of 67.97 M clean reads for each library. More than 10 Gb of high-quality bases were generated for each library. The Q20 value of clean data in each sample was greater than 95%, and the Q30 was greater than 85% (Table S6). The clean reads were mapped to the *Paeonia suffruticosa* Andr. reference genome using Bowtie2 (v2.2.5) software to identify genes corresponding to the clean reads in each library. An average of 81.16% of clean reads were mapped to the reference genome. Clean reads with unique mapping accounted for 8.23% of the reads on average. The RSEM software package was used to evaluate relative abundance values by calculating the fragments per kilobase per million fragments mapped (FPKM). A total of 60,442 transcripts were identified in all 24 samples. The total length of the identified transcripts was 101,830,583 bp. The maximum length observed in transcripts was 8,555 bp, and the minimum length was 273 bp. Among the transcripts, 3,227 (5.34%) were longer than 3000 bp, 55,635 (92.05%) ranged from 500 bp to 3000 bp, and 1,580 (2.61%) were shorter than 500 bp.

### Screening of differentially expressed miRNAs and their predicting target genes

**Figure 5.**
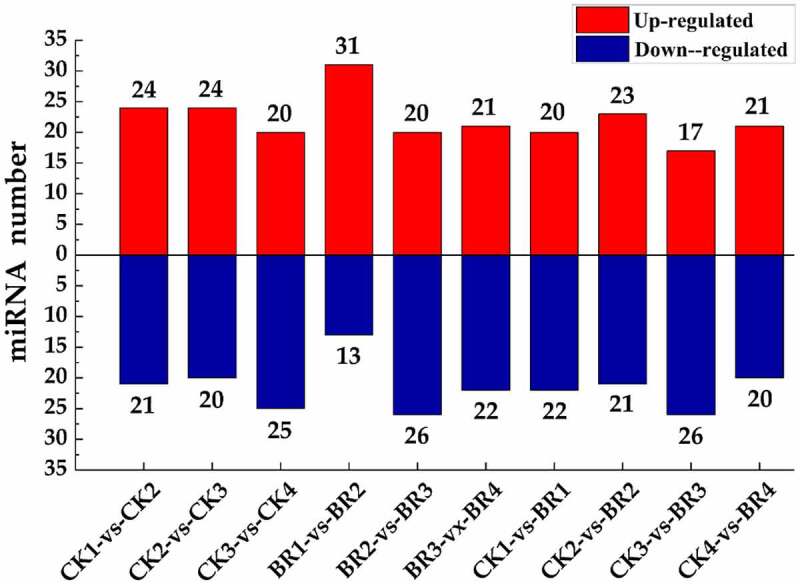
The statistics of differentially expressed miRNA. The X-axis represents different comparison groups. The number on the column chart represents the number of up-regulated or down-regulated differentially expressed miRNAs.

The number of differentially expressed miRNAs based on transcript abundance in the individual samples is shown in Figure 5 and Table S7. The number of differentially expressed miRNAs in each comparison group ranged from 41 to 46. Forty-one differentially expressed miRNAs (21 up-regulated and 20 down-regulated) were identified in the comparison group of BR treatment compared to CK at post-bloom stage in tree peony (CK4-vs-BR4). Forty-six differentially expressed miRNAs (20 up-regulated and 26 down-regulated) were identified in the comparison group of BR treatment at full bloom stage compared to BR treatment at flower opening stage (BR2-vs-BR3).

The target genes of the differentially expressed miRNAs were predicted in each comparison group (Table S8 and Table S9). The number of target genes ranged from 419 (the comparison group of BR treatment compared to CK at bud burst stage, CK1-vs-BR1) to 505 (the comparison group of CK at post-bloom stage compared to CK at full bloom, CK3-vs-CK4). A total of 376 target genes were predicted for the 18 known miRNAs and 177 target genes were predicted for the 23 novel miRNAs. Most of the differentially expressed miRNAs were predicted to have multiple target genes. For example, miR156z, predicted to target 128 genes, had the largest number of target genes identified for a single miRNA. Some of the novel miRNAs only targeted a small number of genes. For example, miR4995, novel_mir56 and novel_mir61 only targeted one gene. One miRNA may target multiple genes, and a single gene might be targeted by one or more miRNAs. Most of the target genes were regulated by only one miRNA, while a few of the identified target genes were targeted by two or three miRNAs.

### GO and KEGG analysis of the target genes

**Figure 6.**
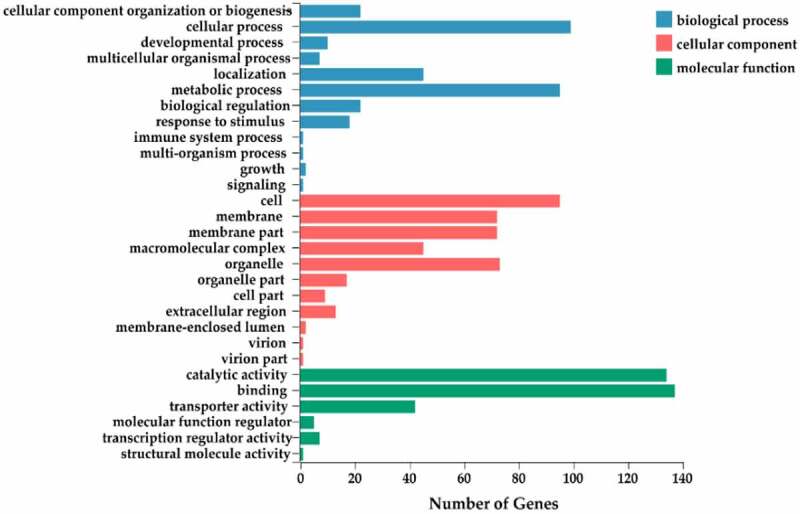
The GO annotation of the target genes.

In order to evaluate the potential function of miRNA target genes, GO and KEGG functional annotations were performed. For comprehensive annotation, all the target genes of differentially expressed miRNAs were analyzed by GO functional annotation. The GO annotations of the target genes were classified into 29 functional groups within the categories of biological processes, cellular components, and molecular function (Figure 6). Within the 12 functional groups enriched in biological process, most of the target genes were categorized under cellular process (GO: 0009987), metabolic process (GO: 0008152), and localization (GO:0051179). Among the 11 functional groups enriched in cellular component, the largest proportion of target genes were categorized under cell (GO: 0005623), followed by organelle (GO: 0043226), membrane (GO: 0016020), membrane part (GO: 0044425), and macromolecular complex (GO: 0032991). Within the six functional groups enriched in molecular function, the dominant categories were binding (GO: 0005488) and catalytic activity (GO: 0003824), followed by transporter activity (GO: 0005215). Further analysis of functional annotation could better understand the important role of target genes in flowering regulation.

**Figure 7.**
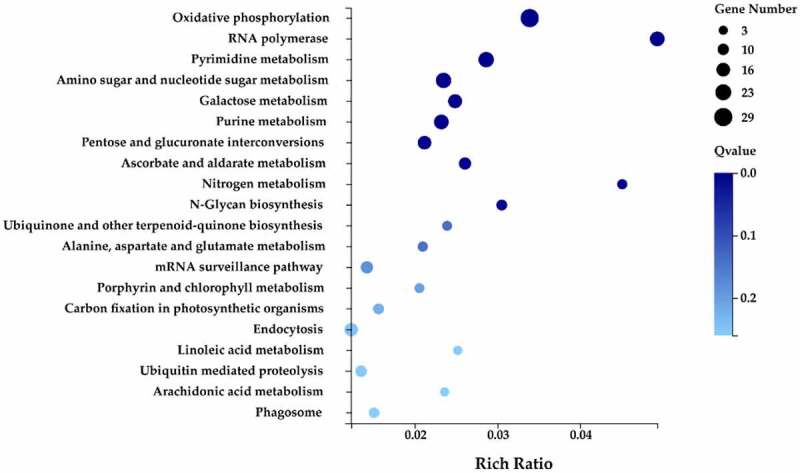
The KEGG annotation of target genes.

Several genes in biological organisms are coordinated and form specific pathways that perform a variety of biological functions. KEGG analysis of genes provides relevant information about their biological function, as the analysis identifies with which pathways a gene is associated. The target genes of the differentially expressed miRNAs were annotated with 18 KEGG terms including five primary categories: cellular processes, environmental information processing, genetic information processing, metabolism, and organism systems (Figure 7). A total of 62 metabolic pathways were identified, of which the number of target genes contained in the oxidative phosphorylation pathway was 29, followed by pyrimidine metabolism (21 target genes) and amino sugar and nucleotide sugar metabolism pathway (21 target genes).

### qRT-PCR analysis of flowering-related miRNAs and their target genes

**Figure 8.**
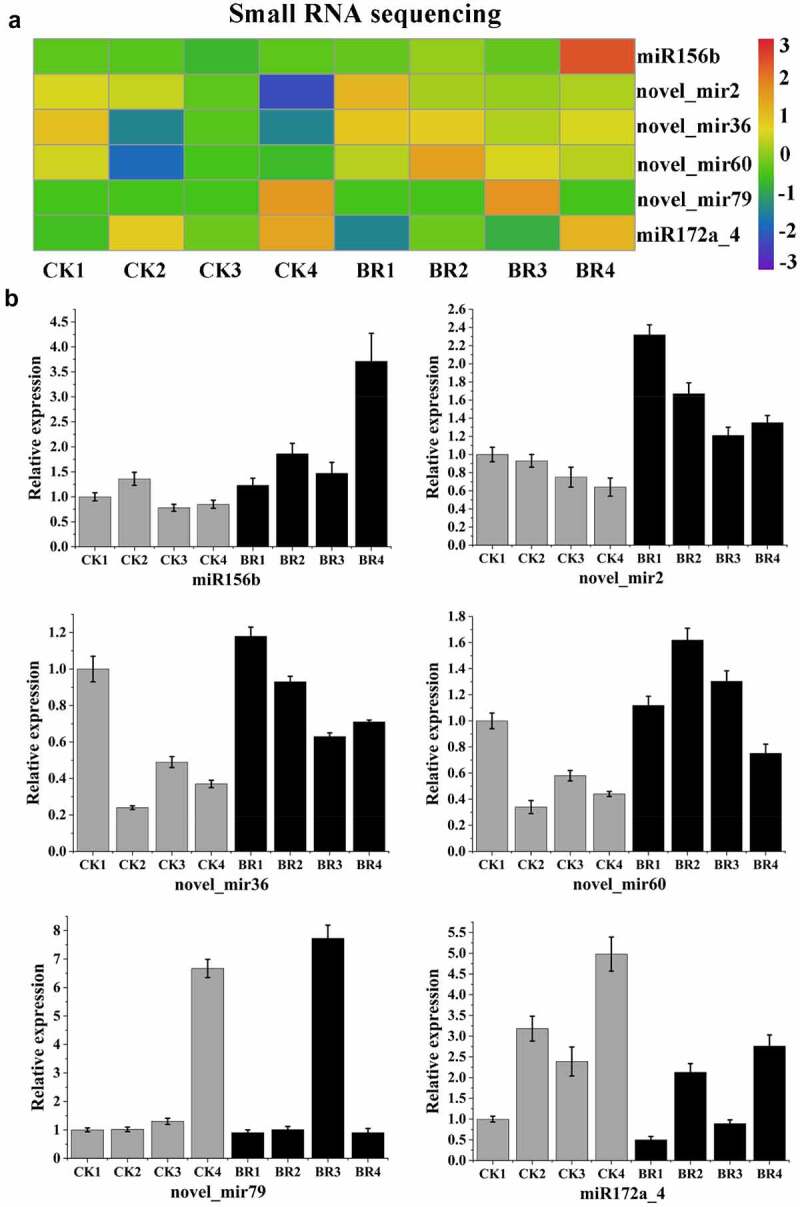
Validation of six differentially expressed miRNAs by qRT-PCR. A, Heatmap and relative expression levels of miRNAs from small RNA sequencing data; B, qPCR expression profile of selected miRNAs.

In order to determine the expression pattern of miRNAs and their target gene response to BR treatment in four continuous developmental stages, the expression of some miRNAs and their target genes that are associated with flowering period was analyzed by qRT-PCR, including six miRNAs (miR156b, miR172a_4, novel_mir2, novel_mir36, novel_mir60, and novel_mir79) (Figure 8). Figure 8 presents the results of the small RNA sequencing data. As expected, qRT-PCR results of six selected miRNAs were consistent with the expression patterns that obtained by small RNA sequencing, and confirmed the changes in miRNA expression in response to BR treatment. After BR treatment, the expression of miR156b, novel_mir2 novel_mir36, novel_mir60, and novel_mir79 increased and the expression of miR172a_4 decreased. Among them, the expression of miR156b, novel_mir36, and novel_mir60 reached a peak in flower-opening stage. The novel_mir2 had the highest expression in bud burst stage. Although the expression trend of novel_mir79 after BR treatment was similar to that of CK data, the highest expression stage shifted. The results of novel_mir79 in CK showed that it had the highest expression level in post-bloom stage, while the data after BR treatment showed that the highest expression period was advanced to full bloom stage. This indicated that BR treatment could promote the expression of novel_mir79.

**Figure 9.**
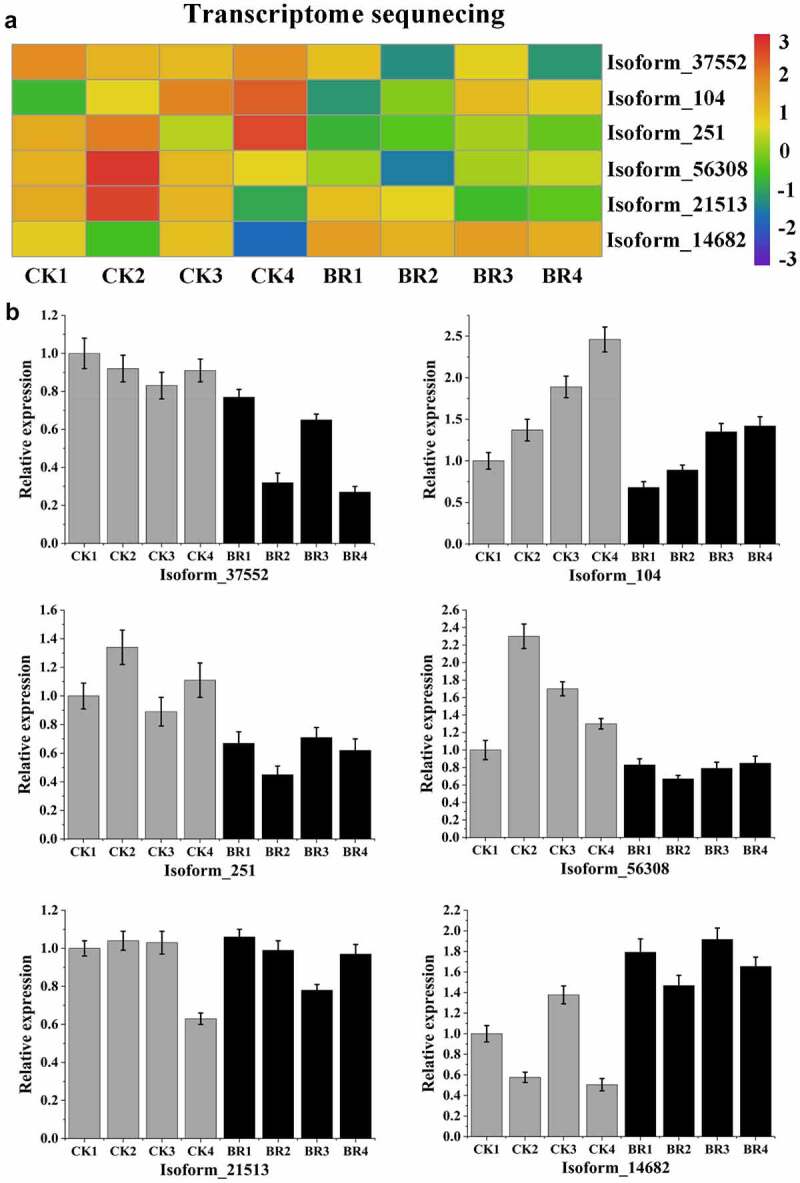
Validation of six target genes by qRT-PCR. A, Heatmap and relative expression levels of target genes from transcriptome sequencing data; B, qPCR expression profile of selected target genes.

Meanwhile, the expression of six target genes, Isoform_37552, Isoform_104, Isoform_251, Isoform_56308, Isoform_21513, and Isoform_14682 were the predicted targets of miR156b, novel_mir2, novel_mir36, novel_mir60, novel_mir79, and miR172a_4, respectively, was also confirmed using qRT-PCR (Figure 9). The qRT-PCR results of these target genes were roughly consistent with the changing trend of transcriptome sequencing, indicating the reliability of sequencing data. The results showed that the expression of these target genes was negatively correlated with the corresponding miRNA.

**Figure 10.**
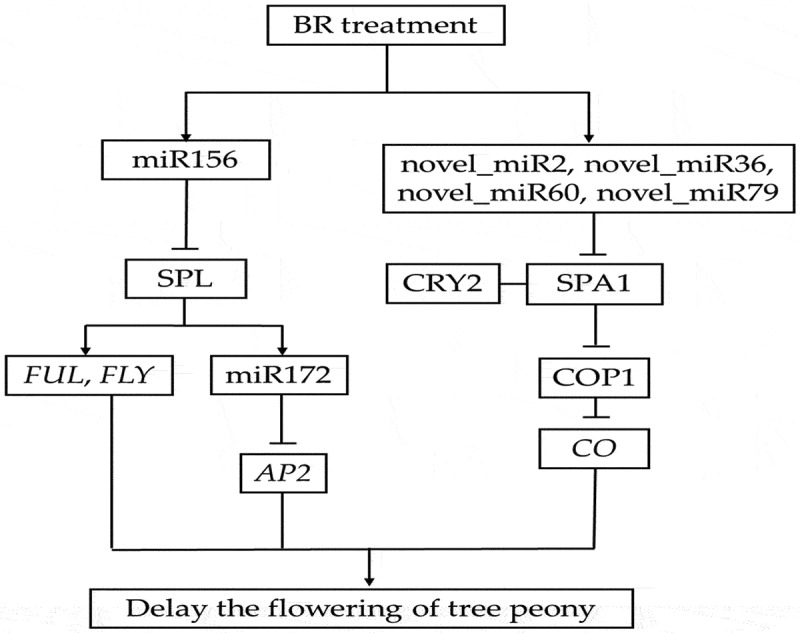
Putative miRNA regulatory network of flowering in tree peony. The line indicates correlation. The arrow indicates facilitation. Bar indicates inhibition.

The integration analysis of miRNA and its target genes showed that BR treatment might inhibit the expression of Isoform_37552, annotating as promoter-binding protein SPL9, by increasing miR156, and further inhibit the expression of *FUL* and *FLY*. Furthermore, the increased expression level of miR156 increases the expression of miR172 and further inhibits the expression of Isoform_14682, annotating as *AP2* gene. On the other hand, it might inhibit the expression of Isoform_104, Isoform_251, Isoform_56308, and Isoform_21513, annotating as *SPA1*, by increasing the expression of novel_mir2, novel_mir36, novel_mir60, and novel_mir79, respectively, so as to inhibit the expression of *CO* (Figure 10). These six miRNAs may be involved in the flowering of tree peony by regulating flowering-related genes in response to BR treatment.

## Discussion

The timing and duration of the flowering period in ornamental plants is one of the important traits contributing to their ornamental and economic value. The ability to regulate the timing and duration of the flowering period could directly affect their prices in the marketplace. Flowering is an important developmental stage in plants as they transition from vegetative to reproductive growth, and it is regulated by a series of gene–environment interactions.^17^ Tree peony is a highly valuable horticultural crop worldwide and has a long history of cultivation and cultural importance in China. But the concentrated and short natural flowering period of tree peony is a direct factor that affects its ornamental value and economic value. BR, as a plant hormone, plays an important role in plant growth and development. There have reported that BR affected the flowering period by postponing its flowering time at Arabidopsis, honeysuckle, and other plants.^11^ But whether BR can affect the flowering period of tree peony has not been reported. Here, we used different concentrations of BR with 25 μg/L, 50 μg/L, 100 μg/L, and 200 μg/L to sprayed petals of tree peony, and founded that the flowering period of tree peony showed varying degrees of flowering delay under the treatment of BR 50 μg/L, 100 μg/L, and 200 μg/L. Especially, at BR 50 μg/L treatment, the flowering period showed obviously delay with 3 days delay that consistent with the results in other species.^12^

Small RNAs are important regulators of biological processes, including plant growth and development, metabolic pathways, and morphogenesis.^34^ Although miRNAs have been identified in tree peony roots, stems, leaves, flower buds, and seeds using small RNA-seq,^30,31^ the mechanism of flowering time in tree peony is still unclear. In the present study, the raw read count in independent samples between 25.0 and 61.5 million reads was achieved by using small RNA sequencing. The type of small RNAs were determined by their characteristically length distribution ranged from 18 to 30 nt. The lengths of the small RNA sequences (21–24 nt, witch 24 nt miRNAs were most abundant) is consistent with previous reports in tree peony seeds,^32^ *Arabidopsis*,^35^ and rice (*Oryza sativa*).^36^ In addition, the analysis of the first nucleotide of the miRNAs revealed that many miRNAs start with U at the 5’ end, which is also consistent with a previous report of miRNAs in tree peony seeds.^32^ There have been few reports on small RNAs in tree peony. A total of 318 known miRNAs and 153 novel miRNAs were previously identified in tree peony seeds.^32^ Additionally, 89 known miRNA families and 34 newly predicted miRNAs were identified in tree peony vegetative tissues in response to copper stress.^30^ In our present study, a total of 22 known miRNAs and 84 novel miRNAs were identified. This information can serve as a foundation for further studies on the role of miRNAs and their target genes on the regulation of flower development in tree peony.

Transcriptome analysis is the foundation for studying gene function and elucidating the molecular mechanisms underlying specific biological processes. A normalized cDNA library was constructed from mixed petals at different developmental stages in ‘Luoyang Hong’ tree peony, and obtained approximately 4.8 Gb of high-quality nucleotides.^37^ Subsequently, transcriptome analysis was carried out on flower petals of ‘Luoyang Hong’ tree peony in closed buds and at full bloom, and more than 8 Gb clean data were obtained.^38^ In the present study, the amount of sequence data obtained was significantly more than that obtained in the previous studies of tree peony. The total of 60,442 transcripts were also identified in the present study, which was again significantly more than the number of transcripts obtained in previous flowering studies in tree peony.^5,37,38^ Therefore, the transcriptome resource obtained in our study much improves the available transcript data for tree peony and provides genetic information for future studies.

miRNAs can negatively regulate target gene expression through complete or partial complementary pairing with target mRNA or inhibition of protein translation. In this study, several differentially expressed miRNAs were identified in every group comparison in our analysis. During the flowering process in tree peony, a total of 18 known miRNAs and 53 novel RNAs were detected and were found to be differentially expressed during the period of flowering analyzed in the current study. miR156^39^ and miRNA172,^40^ related to the regulation of plant flowering, were detected in the present study and found to be differentially expressed between different stages of petal development. Additionally, some potential flower regulatory miRNAs, including miR171, miR172, miR397, miR399, miR4995, miR5141, miR530, miR5532, and miR845, were also detected in the current study. The identification of the target genes of miRNAs can provide important information on the potential role of miRNAs in regulating growth and development. Among the differentially expressed miRNAs identified in our study, 376 target genes were predicted for the 18 known miRNAs, and 177 target genes were predicted for the 23 novel miRNAs. Among them, miR156z was predicted to have the greatest number of target genes (128 target genes), which may reflect the high-level of conservation of miRNA156. This result was consistent with previous reports in tree peony seeds,^32^ rice,^41^ and wheat,^42,43^ where miRNA156 always had the largest number of predicted target genes. Some of the novel miRNAs identified, however, were predicted to have only a small number of target genes. This may be due, however, to the lack of data on tree peony small RNAs in the miRBase database.

Based on the results of the GO classification conducted in this study, cell process, cell, and binding were the functional groups within the primary category of biological processes with the largest number of target genes. In a previous study of flower petals at different developmental stages in ‘Luoyang Hong’ tree peony, a large number of target genes were also classified in cell process, cell, and binding functional groups.^37^ Therefore, our results are consistent with this previous study. GO annotation of a sequenced cDNA library of mixed buds in tree peony resulted in the classification of a large number of genes in metabolic processes, regulation of transcription, and binding.^31^ These results in this study were somewhat different from the previously mentioned study,^31^ which may have been due to the sampling of different tissues in the different studies. Moreover, the composition of the transcriptome is dynamic and is affected by development stage and process, environmental signals, and stress.^41^ A subsequent KEGG pathway analysis revealed that most of the target genes in all of the group comparisons were significantly enriched in the metabolism category, primarily in carbohydrate metabolism, energy metabolism, and biosynthesis of other secondary metabolites. The GO classification and KEGG pathway enrichment analyses collectively provide important information that can be used to develop a theoretical basis for the regulation of flowering in tree peony and serve as a foundation for future studies.

In the present study, six small RNAs and their corresponding six target genes were identified that may potentially be related to the regulation of flowering period in tree peony. Among them, Isoform_37552 targeted by miRNA156b in the present study was homologous to *SPL*, and Isoform_14682 targeted by miR172a_4 was homologous to *AP2. SPL* is a plant-specific transcription factor that regulates a variety of developmental processes^44^ and is primarily regulated by miRNA156. miRNA156 is composed of approximately 20 nucleotides, has a conserved structure, and functions in the regulation of growth and flowering by targeting the *SPL* family of transcription factors.^45^ miRNA156 inhibits the expression of *SPL* by shearing degradation and inhibiting the translation of *SPL* mRNA.^29,46^

At present, miR156-*SPL* module has been found to affect the flowering process of plants mainly by regulating downstream flowering-related genes. At the stem tip of *Arabidopsis thaliana, AtSPL3/4/5* and *AtSPL9* can directly activate downstream MADS-box genes such as *LEAFY (LFY), FRUITFULL (FUL), AGAMOUSLIKE (AGL42)*, and *AP1* to promote flowering.^47^ In the leaves of *Arabidopsis thaliana, AtSPL9* reduces the expression level of downstream *AP2-like* flowering suppressor genes by activating miR172, forming a miR156/157-*SPLs*-miR172-*AP2-like* regulatory pathway, thereby promoting flowering.^48,49^ In addition, DELLA protein, a transcription inhibitor in the gibberellin pathway, can reduce the activity of *SPL* by interacting with the target gene *SPL* of miR156. On the one hand, delayed flowering can be induced by lowering the expression level of downstream MADS-box flowering genes under short-day conditions. On the other hand, the miR172-*AP2-like* pathway inhibits the expression of flowering gene *FT*, resulting in delayed flowering under long sunshine.^50^ At the same time, *AtSPL15* also relies on GA signaling to recruit MED18 and RNA polymerase II, guiding downstream *FUL* and miR172 to participate in the flowering process.^40^ In addition to being involved in plant age regulation, miR156-*SPL* also responds to vernalization. Expression of the *TOE1* gene in the *AP2-like* family causes *Cardamine flexuosa* to be insensitive to low temperatures and unable to vernalize and bloom normally. *TOE1* is positively regulated by miR156, and the expression of *TOE1* and miR156 decreases with the progress of plant growth, thus improving the low-temperature sensitivity of *Camelina camelina* and inducing flowering.^51^ These results suggest that miR156-*SPL* is not only an important factor in age-regulating flowering pathways but also synergistically regulates flowering processes by integrating GA and vernalization pathways. In this study, after BR spraying, miR156k expression increased, while miR172a_4 expression decreased, which was consistent with previous theoretical research results.

Four of the genes, targeted by four novel miRNAs, were putatively annotated to SUPPRESSOR OF PHYTOCHROME A-105 1 (*SPA1*). *SPA1* is one of the important regulatory factors in the regulation of light signal transduction pathway.^52^ The blue light receptor CRY is an important photoreceptor in the model plant *Arabidopsis thaliana*, which regulates important physiological processes such as photomorphogenesis, flowering time, biological rhythm, stomata opening, and stomata development in plants.^53^ The main function of CRY1 is to inhibit hypocotyl elongation, while CRY2 mainly regulates the flowering initiation induced by photoperiod. CRY2 can interact with SPA1 in a blue light-dependent manner to inhibit the degradation of CO (CONSTANS) by the COP1 (CONSTITUTIVEPHOTOMORPHOGENIC1)-SPA1 complex, thereby stabilizing CO, and ultimately controlling the initiation of flowering by regulating the transcription of downstream target gene *FT*.^53^ CRY1 and CRY2 regulate the expression of miRNA172 under blue light to adjust flowering time.^24^ In our study, the expression levels of novel_mir2 and novel_mir36 increased after BR treatment. And the flowering of ‘Feng Dan’ was delayed by 3 days in response to the BR treatment. This result is consistent with previous theoretical research results. The specific molecular mechanism of this plausible scenario, however, remains to be further studied.

## Conclusions

In this study, we explored the effect of BR on the flowering period of peony by different concentrations of BR treatment and found that the flowering period of tree peony showed visibly delay at BR 50 μg/L treatment. A total of 22 known miRNAs and 84 novel miRNAs were identified and predicted via small RNA sequencing and transcriptome sequencing. Among the 106 significantly changed miRNAs, six miRNAs and their target genes were found to be possibly involved in regulating flowering period, including four novel miRNAs targeting *SPA1*, miR156b targeting *SPL*, and miR172 targeting *AP2*. This study would provide a theoretical basis for understanding the role of small RNA in regulating flowering time in tree peony. This work provides the small RNA and their target genes expression analysis in tree peony, which offers critical clues to reveal the molecular mechanisms in response to BR treatment on flowering of tree peony.

## Supplementary Material

Supplemental Material
